# Educational Inequalities in Perinatal Outcomes: The Mediating Effect of Smoking and Environmental Tobacco Exposure

**DOI:** 10.1371/journal.pone.0037002

**Published:** 2012-05-10

**Authors:** Gerrit van den Berg, Manon van Eijsden, Tanja G. M. Vrijkotte, Reinoud J. B. J. Gemke

**Affiliations:** 1 Department of Pediatrics, VU University Medical Center, Amsterdam, The Netherlands; 2 Department of Epidemiology, Documentation and Health Promotion, Public Health Service, Amsterdam, The Netherlands; 3 Department of Health Sciences, VU University, Amsterdam, The Netherlands; 4 Department of Public Health, Academic Medical Center, University of Amsterdam, Amsterdam, The Netherlands; Emory University School of Medicine, United States of America

## Abstract

**Objective:**

Socioeconomic status (SES) is adversely associated with perinatal outcomes. This association is likely to be mediated by tobacco exposure. However, previous studies were limited to single perinatal outcomes and devoted no attention to environmental tobacco exposure. Therefore, this study aimed firstly to explain the role of maternal smoking in the association between maternal education and preterm birth (PTB), low birth weight (LBW) and small for gestational age (SGA), and secondly to explain whether environmental tobacco smoke mediates these associations further.

**Study Design:**

This study was nested in a population-based cohort study in the Netherlands, the Amsterdam Born Children and their Development (ABCD) study. Analyses were done in a sample of 3821 pregnant women of Dutch origin, using logistic regression analysis.

**Results:**

Least educated women, who were more often smoking and exposed to environmental tobacco smoke, had a significantly higher risk of PTB (OR 1.95 [95% CI: 1.19–3.20]), LBW (OR 2.41 [95% CI: 1.36–4.27]) and SGA (OR 1.90 [95% CI 1.32–2.74]) than highly educated women. The mediating effect of smoking in the least educated women was 43% for PTB, 55% for LBW and 66% for SGA. Environmental tobacco smoke did not explain these associations further. After adjustment for maternal smoking, the association between lower maternal education and pregnancy outcomes was no longer significant.

**Conclusions:**

Smoking explains to a considerable extent the association between lower maternal education and adverse perinatal outcomes. Therefore, tobacco-interventions in lower educated women should be primarily focussed on maternal smoking to reduce PTB, LBW, and SGA. Additional attention to environmental tobacco exposure does not seem to reduce educational inequalities in perinatal outcomes.

## Introduction

Adverse perinatal outcomes, such as preterm birth (PTB), low birth weight (LBW), and small for gestational age (SGA), are strongly related to neonatal morbidity as well as future adult morbidity. More specifically, PTB may result in ophthalmologic, pulmonary, cognitive, behavioural or emotional problems [1], while LBW and SGA may increase the risk of cardiovascular disease, type 2 diabetes and psychomotor and intellectual impairment [2], [3]. Moreover, all these adverse perinatal outcomes lead to increased perinatal mortality [4].

Perinatal morbidity has been associated with socioeconomic status (SES) [5]–[7]. For instance, compared to high SES women, women of low SES are more likely to give birth prematurely [7]–[10], have low birth weight [7], [11], [12], and small for gestational age offspring [7], [13]–[15]. More specifically, it must be noted that even in an affluent society with a high level of social security, relatively small income differences and easy access to medical care, low social class has been associated with reduced birth weight and an increased frequency of PTB [16], [17]. Although various investigators have reported that after adjustment for known confounding factors, socioeconomic status may not be an important independent contributor to perinatal outcomes [18], socioeconomic disparities in perinatal outcomes have not been explained adequately so far [6]. A lower SES has no direct effect on adverse perinatal outcomes; rather it may be associated with adverse risk behaviour, such as greater tobacco exposure.

Tobacco exposure appears to be a strong biologically plausible mediator of socioeconomic differences in perinatal outcomes. It is well-known that tobacco exposure is responsible for an etiologic fraction of the adverse pregnancy outcome [6], [19]. Causality has been implicated by repeated observations of a dose-response relationship [19]–[22], and by a possible maternal metabolic gene that modified the association between maternal cigarette smoking and infant birth weight [23]. In addition, tobacco exposure is strongly associated with SES [24], [25]. For example, tobacco exposure is more prevalent among lower educated women [26]. Various studies have demonstrated the role of tobacco exposure in the relation between LBW or SGA and SES, which was estimated at 45–66% for LBW and 38–47% for SGA [15], [27]. Other studies have suggested that tobacco exposure mediates the relation between maternal education and PTB [6], [10]. Besides maternal smoke, environmental tobacco exposure (ETE) might play an additional role in the relation between socioeconomic status and adverse perinatal outcomes. In a meta-analysis ETE appears to be associated with LBW and SGA although effects are generally smaller than those for maternal smoking and in most studies not statistically significant.[28] More recently, ETE did not affect mean birth weight significantly [29], but others found specific associations with severe SGA [30]. ETE is associated with socioeconomic status as well [31], but as far as we know the contribution of ETE on socioeconomic disparities in perinatal outcomes has not been examined previously.

So far studies on the explanatory role of tobacco exposure were each limited to one single perinatal outcome. A key problem is that the explanatory role of tobacco exposure cannot be compared between different perinatal outcomes, because most studies assessed different measures of SES and added different covariates. As far as we know, only Gissler et al. explained the role of tobacco exposure in educational inequalities in more than one perinatal outcome, but devoted no attention to ETE [27].

To better understand the mediating effect of tobacco exposure on the socioeconomic disparities in perinatal outcomes, this current study aimed (i) to investigate whether maternal smoking mediates the educational inequalities in three main perinatal outcomes and (ii) to assess whether there is additional mediation by ETE. The perinatal outcomes that were investigated were PTB, LBW, and SGA. This study was conducted in a large population-based cohort and involved ethnic Dutch participants only, as educational inequalities in pregnancy outcome may differ between Dutch women and women with another ethnic background [6], [16].

## Methods

The present study is part of the Amsterdam Born Children and their Development (ABCD) study, a prospective, longitudinal birth cohort. Details of the study were described previously [32]. In brief, all pregnant women in Amsterdam, the Netherlands, were invited to participate at their first antenatal visit with their obstetric caregiver between January 2003 and March 2004. In total 12 373 women were invited and 8266 women were enrolled in the study by returning the pregnancy questionnaire (response rate 67%) at a gestational age of 16.05 weeks (SD 3.8). These data were completed with information on pregnancy outcomes from Youth Health Care Registration and the Dutch Perinatal Registration (PRN). Birth weight and gestational age did not differ between respondents and non-respondents [33]. We excluded twin pregnancies (n = 135). Participants with missing data on education (n = 69) were excluded as well. In order to exclude potential ethnic confounding we only involved ethnic Dutch participants (i.e first and second generation immigrants were excluded (n = 4148)). Participants with missing data on gestational age (n = 40), birth weight (n = 10), tobacco exposure (n = 1), those with a non spontaneous abortion (n = 9) or birth after a gestation of <24 weeks (n = 33) were excluded as well, so finally there were 3821 participants in the study population. Approval was obtained from the VU University Medical Center Medical Ethical Committee, Academic Medical Center Medical Ethical Committee, and the Registration Committee of Amsterdam. All participating mothers gave written informed consent.

### Main variables

The number of years of education after primary school was obtained by questionnaire, and categorized as low (less than 6 years of education after primary school), mid (6 to 10 years) and high (more than 10 years). Education is the most frequent used single indicator of SES and typically measured as years completed [34].

Three major perinatal outcomes were explored, i.e. PTB, LBW, and SGA. PTB was defined as a delivery from 24 0/7 through 36 6/7 weeks of gestation. Data on gestational duration were based on ultrasound or, when unavailable (<10%), on timing of last menstrual period. LBW was defined as a weight below 2500 grams. Newborns were categorized as SGA if they had a birth weight below the 10^th^ percentile for gestational age on the basis of sex- and parity- specific standards from the Netherlands [35].

Smoking and environmental tobacco exposure during pregnancy were self-reported in the pregnancy questionnaire. Smoking was categorized into four groups: nonsmoking and no ETE, nonsmoking and ETE (≥1 cigarette a day), smoking (≥1 cigarette a day) and no ETE, and smoking and ETE.

### Covariables

The following covariables were measured in the pregnancy questionnaire and/or the perinatal registry and were included in the analyses: sex, maternal age, maternal height, parity (0, ≥1), maternal pre-pregnancy body mass index (BMI; kg/m^2^)

### Statistics

Differences in general characteristics between women with low, mid, and high educational level were tested with ANOVA analysis for continuous variables and Chi-square tests for categorical variables. Firstly, the association between tobacco exposure and perinatal outcomes was examined using logistic regression analyses (reference group: nonsmoking and no ETE; additional reference group: smoking and no ETE). Secondly, univariate analyses were conducted for the association between maternal education (reference group: high) and perinatal outcomes, followed by multivariable analyses, including all relevant covariates simultaneously (model 1). Maternal height (linear) was included as a continuous variable, maternal age and maternal BMI were included as categorical variables. For SGA analysis, sex and parity were excluded because the definition of SGA already accounts for these covariates. Finally, to investigate the mediating effect of tobacco exposure, smoking and environmental tobacco exposure were added to the full multivariable model additionally (model 2 and 3).

To test the quantitative effect of smoking on top of the fact whether or not women were smoking, we tested in the subgroup of smoking women whether the number of cigarettes which were smoked differed between women with PTB, LBW, or SGA offspring and those without PTB, LBW, or SGA offspring respectively using an independent sample t-test. The same was done for ETE.

The percentage change in odds ratio due to adding tobacco exposure to the model was calculated with the formula: ([OR_model1_−OR_model + smoking_]/[1−OR_model1_] * 100), provided that the adjusted model (model 1) showed a significant association.

All statistical analyses were performed using the Statistical Package of Social Sciences version 15.0 for Windows (SPSS Inc., Chicago, IL, USA). A *p*-value<0.05 was regarded as significant in all analyses.

## Results

Table 1 shows the general characteristics of the study sample by maternal educational level. The 316 (8.3%) women with a lower educational level were significantly younger, had a shorter height, were less often primiparous and had a higher BMI than the 56,4% of women with a higher educational level.

**Table 1:** General characteristics according to maternal educational level.

		Educational level			
	Total (n = 3821)	Low (n = 316)	Mid (n = 1348)	High (n = 2157)	p-value
**Pregnancy characteristics**					
Maternal age					<.001
*<25 years (%)*	5.0	24.1	7.6	0.7	
*25–34 years*	67.2	52.5	64.2	71.2	
*≥35 years*	27.8	23.4	28.2	28.1	
Maternal height, mean (SD)	171.47 (6.20)	169.81 (6.36)	171.13 (6.50)	171.93 (5.93)	<.001
BMI (kg/m2) (%)					<.001
*<18.5*	4.1	6.6	3.7	3.9	
*18.5–25*	79.5	65.5	75.9	83.8	
*>25*	16.4	27.8	20.4	12.2	
Parity (% primipara)	60.1	53.5	61.2	60.4	.04
Infant sex (% boys)	50.2	53.5	48.6	50.8	.21
Tobacco exposure					<.001
*Nonsmoking and no ETE*	79.0	44.6	73.8	87.2	
*Nonsmoking and ETE*	13.8	22.2	17.0	10.6	
*Smoking and no ETE*	1.5	6.3	1.5	0.8	
*Smoking and ETE*	5.7	26.9	7.7	1.4	
Number of cigarettes a day, mean (SD)^1^	7.1 (4.9)	8.9 (6.0)	6.6 (3.9)	4.6 (3.1)	<.001
Number of cigarettes a day exposed to, mean (SD)^2^	7.0 (7.1)	10.4 (7.4)	6.8 (7.5)	5.4 (5.8)	<.001
**Outcome**					
Prematurity (%)	4.9	7.6	4.9	4.5	.05
Low birth weight (%)	3.6	5.7	4.5	2.6	.001
Small for gestational age (%)	9.4	15.5	9.4	8.5	<.001

Significance values are based on Chi-square Tests. Height was based on one-way ANOVA analysis. ETE indicates environmental tobacco exposure.

1 subgroup of smoking women (n = 277).

2 subgroup of women who were exposed to environmental tobacco (n = 746).

### Tobacco exposure

As can be seen from Table 1, tobacco exposure, both smoking and environmental tobacco smoke, was more frequent in lower educated women. If the women smoked or were exposed to environmental tobacco, the number of cigarettes was higher among lower educated women compared to higher educated women. ETE was not related to adverse perinatal outcomes, whereas smoking was. The odds ratios for each perinatal outcome are presented in Table 2. The amount of cigarettes smoked each day was reported equally between women who were exposed to environmental tobacco smoke and those who were not.

**Table 2:** Univariate logistic regression analysis of tobacco exposure and perinatal outcomes.

	Tobacco exposure			
	Nonsmoking and no ETE	Nonsmoking and ETE	Smoking and no ETE	Smoking and ETE
		OR (95% CI)	OR (95% CI)	OR (95% CI)
n	3017	527	58	219
Preterm birth	Reference	0.91 (0.57, 1.44)	1.97 (0.77, 5.00)	2.21 (1.37, 3.58)
Low birth weight	Reference	0.90 (0.52, 1.57)	2.25 (1.15, 4.40)	3.09 (1.87, 5.11)
Small for gestational age	Reference	1.05 (0.76, 1.46)	2.86 (1.50, 5.47)	3.16 (2.24, 4.46)
Preterm birth			Reference	1.12 (0.41, 3.12)
Low birth weight			Reference	1.07 (0.38, 2.97)
Small for gestational age			Reference	1.11 (0.54, 2.25)
Cigarettes per day, mean (sd)§	None	None	6.7 (4.4)	7.2 (5.1)

§ no significant difference with one way ANOVA-analysis. ETE indicates environmental tobacco exposure.

### Educational inequalities

In the present study, the prevalence of adverse perinatal outcomes was higher among the lower educated group compared to the higher educated group. The lower educated women had a significantly increased risk for preterm birth (OR 1.95 [95% CI 1.19–3.20]), low birth weight (OR 2.41 [95% CI 1.36–4.27]) and SGA birth (OR 1.90 [95% CI 1.32–2.74]) (Table 3, adjusted model).

**Table 3:** Associations between maternal education and perinatal outcomes.

	Educational level	Crude model	Model 1	Model 2	Model 3
		OR (95% CI)	OR (95% CI)	OR (95% CI)	OR (95% CI)
PTB	Low (n = 24/316)	1.77 (1.11, 2.81)	1.95 (1.19, 3.20)	1.54 (0.90, 2.62)	1.58 (0.93, 2.69)
	Mid (n = 66/1348)	1.10 (0.80, 1.52)	1.11 (0.80, 1.54)	1.05 (0.76, 1.47)	1.07 (0.77, 1.49)
	High (n = 96/2157)	Reference	Reference	Reference	Reference
LBW	Low (n = 18/316)	2.23 (1.29, 3.83)	2.41 (1.36, 4.27)	1.64 (0.88, 3.04)	1.69 (0.91, 3.14)
	Mid (n = 61/1348)	1.75 (1.21, 2.52)	1.73 (1.19, 2.51)	1.56 (1.07, 2.29)	1.60 (1.09, 2.35)
	High (n = 57/2157)	Reference	Reference	Reference	Reference
SGA	Low (n = 49/316)	1.97 (1.40, 2.77)	1.90 (1.32, 2.74)	1.31 (0.88, 1.95)	1.30 (0.87, 1.93)
	Mid (n = 127/1348)	1.12 (0.89, 1.42)	1.10 (0.87, 1.41)	1.00 (0.78, 1.28)	0.99 (0.77, 1.27)
	High (n = 184/2157)	Reference	Reference	Reference	Reference

PTB indicates preterm birth, LBW indicates low birth weight, SGA indicates small for gestational age. Model 1: Adjusted for sex, maternal age (categorical), height, parity, pre-pregnancy BMI (categorical). SGA analysis, exclusion of parity and sex. Model 2: Model 1 adjusted for smoking (yes/no). Model 3: Model 2 adjusted for environmental tobacco exposure (yes/no).

### Mediating effect of tobacco exposure

Smoking explained about 43% of the association between lower education and PTB, 55% of the association between lower education and LBW and 66% of the association between lower education and SGA. (Table 3, model 2). In mid-educated women, smoking explained 24% of educational inequalities in LBW. Additional adjustment for ETE did not decrease the association between maternal education and perinatal outcomes further. After adjustment for tobacco exposure, the association between maternal education and adverse perinatal outcomes was no longer significant, except for LBW in mid educated women (OR 1.60 [95% CI 1.09–2.35]).

As smoking women with a PTB infant on average smoked more cigarettes a day compared to smoking women with a term born infant (9.4 vs 6.9, p = 0.01), it was expected that adjustment of the number of cigarettes on top of the dichotomous variable (smoking yes/no), might explain the association between maternal education and PTB further. As there were only 26 PTB infants among smoking women we were however not able to test this. The number of smoked cigarettes did not differ between smoking women with an LBW or SGA infant and those without LBW or SGA infant. Thus, there was no indication that the number of cigarettes could further explain the association between maternal education and SGA or LBW. As there was also no association between the number of environmental cigarettes exposed to and PTB, LBW, and SGA in the environmentally exposed subgroup, the same applies to ETE.

## Discussion

This study regarding the mediating role of tobacco exposure on educational inequalities in three main perinatal outcomes found that in general, higher rates of preterm birth, low birth weight, and small for gestational age, were present among women with lower education. Smoking is largely responsible for education related differences in perinatal outcomes, while there seems no additional role for environmental tobacco exposure.

### Comparison with other studies

Consistent with previous studies, an association of socioeconomic status was found with PTB [7], LBW [11], and SGA birth [8]. Of the participating women 7.2% reported smoking, which is a slightly lower prevalence than others described, most likely due to the exclusion of ethnic minorities, and a possible underreporting of smoking. Our final sample also might have a lower prevalence of smoking due to selection bias. For example, the participation rate declined with lower income (based on neighbourhood-income) and women with a birth below 24 weeks of gestation, who reported a higher prevalence of smoking, were excluded [33], [36]. Smoking was associated with PTB, LBW, and SGA, which corresponds with other studies [19], [22], [27]. Although it seems that the odds of adverse perinatal outcomes increases if the mother is exposed to environmental tobacco smoke in addition to smoking, there was no significant association between environmental tobacco smoke and adverse perinatal outcomes. In a review, Misra et al. [28] reported for example, that in three of the six studies, the odds of LBW were significantly and substantially increased for infants born to women exposed to ETE, although in two of the studies the significant effect was only demonstrated in a subgroup of the women. These inconsistencies may be due to differences in the method of determining exposure to ETE, and the timing of ETE exposure, because the prevalence of ETE might decrease during pregnancy when others know that the woman is pregnant.

### Smoking

In the present study, smoking explains the association between maternal education and perinatal outcomes to a considerable extent, since this decreased association ranged from 43% to 66% for various perinatal outcomes. To our knowledge, only Gissler et al. described the role of smoking in the association between socioeconomic status and various perinatal outcomes [27], but the role of smoking is found to be lower than in our study. In addition, Beard et al. found that about 40% of the relation between socioeconomic disadvantage and SGA is explained by smoking [15]. Lower percentages than we found might be firstly because we initially corrected for possible confounders and used therefore a better method for estimating the mediating role of smoking, and secondly because we used another measure for SES. Among smoking women in our study, the number of smoked cigarettes was associated with PTB, so additional adjustment for the number of smoked cigarettes might decrease the association between maternal education and PTB. As with PTB [37], there is evidence for a dose-dependent relationship between smoking and SGA [18], though we could not prove this in our study. ETE appears not to explain educational inequalities in perinatal outcomes further, most likely because the effect of ETE is negligible compared to smoking.

### Pathophysiological mechanism

Previous studies have reported several mechanisms linking tobacco exposure to adverse perinatal outcomes. First, tobacco exposure may lead to impaired fetal oxygen delivery due to a reduction in the fraction of capillary volume in the placenta and an increased thickness of the villous membrane [38], and smoking decreases acute intervillous perfusion as well [39]. Second, carbon monoxide exposure from cigarettes may lead to carboxyhemoglobin formation, which diminishes fetal tissue oxygenation. Third, smoking may cause direct damage to fetal genetic material, which can lead to chromosomal abnormalities in particular [40]. Although, these three factors are well established, there may be other injurious effects of cigarette smoking, such as toxicity of other chemicals in mainstream tobacco smoke and the sympathetic activation leading to acceleration of fetal heart rate and a reduction in fetal breathing movements. As these mechanisms retard intra-uterine growth, these may be more likely to affect birth weight instead of preterm birth, which is supported by our results. However, there is a strong interaction between preterm birth and low birth weight and between each of these two variables and intrauterine growth retardation and it is not easy to disentangle the effects.

### Strengths and limitations

Firstly, the major strengths of the present study are the population-based sample, the prospective study design, the comparison of multiple outcomes, and, moreover, the fact that environmental tobacco smoke was taken into account. As in all cohort studies, these strengths were limited by possible selection bias. As mentioned before, participants were more likely to live in a higher income neighbourhood. However, it is described earlier that birth weight and gestational age did not differ between participants and non-participants, and we can think of no reasons why the associations we have examined here should be markedly different in non-participants [33]. Furthermore, a limitation is the fact that tobacco exposure was measured at a single time point only, namely early pregnancy. Although infant growth is greatest in last trimester and tobacco exposure might influence infant growth especially during that pregnancy period, it was assumed that women still smoking after their first prenatal visit are likely to continue smoking during pregnancy. In a Swedish study 32% of the pregnant women reported smoking at the time of conception, 18% stopped prior to the first prenatal visit, 7% between 10 and 24 weeks, and 4% in the third trimester [41]. Because quitting rates were lower among women at low socioeconomic status [42], we infer that our results will probably be an underestimation of the impact on the population. ETE exposure might also change over the course of pregnancy. Women may stop working as their pregnancy progresses and co-workers might reduce their smoking around a woman as she becomes visibly pregnant [28]. Future studies may benefit from repeated measurements of tobacco exposure. Secondly, our study relied on self-report to assess exposure to environmental tobacco. Social disapproval of smoking may influence the truthfulness of the women's response to the smoking-questions so attenuation of the role of tobacco exposure could occur. It was shown that self-reported smoking correlates well with serum cotinine levels [43], [44], but the self-reported ETE was less reliable [44]. In particular for ETE, a biomarker assessment of environmental tobacco exposure may be more valid than self-reported exposure because it can also account for differences in exposure that are not captured by reporting the number of cigarettes one is exposed to [28]. For example, differences in hours of exposure, and ventilation are ignored by self-reported assessment. However, epidemiologic studies of smoking and adverse pregnancy outcomes typically rely on maternal self-report [45]. Thirdly, because of a non-normal distribution maternal cigarette smoking and environmental tobacco exposure were dichotomized. This could underestimate the contribution of smoking to inequalities in perinatal outcomes. As far as possible, we had no indication that adding the number of cigarettes has an additional effect. Finally, the strength was the homogeneity of the sample, which only comprised women of Dutch ethnicity. Therefore, educational inequalities were not obscured by ethnicity. For further research we recommend investigating whether tobacco exposure is responsible for the relation between perinatal outcomes and other components of SES, such as income and occupation, because maternal education does not cover the entire SES, although this is an important proxy of SES.

### Conclusion

In conclusion, this study demonstrated that maternal smoking during pregnancy to a considerable extent is responsible for the association between relatively low maternal education and preterm birth, and to a markedly extent to low birth weight, and small for gestational age. Environmental tobacco exposure did not seem to have an additional role in explaining these associations. While eliminating smoking is of public interest, smoking still appears to contribute to adverse perinatal outcomes in lower educated women. These findings indicate that tobacco interventions in order to reduce adverse perinatal outcomes in lower educated women should be primarily focused on smoking itself and not be directed to environmental tobacco exposure.
